# Effect of Heat-Killed *Lactobacillus casei* DKGF7 on a Rat Model of Irritable Bowel Syndrome

**DOI:** 10.3390/nu13020568

**Published:** 2021-02-09

**Authors:** Gyeol Seong, Seungbaek Lee, Yang Won Min, Yeon Sil Jang, Hong Seog Kim, Eui-Joong Kim, So-Young Park, Cheol-Hyun Kim, Dong Kyung Chang

**Affiliations:** 1 Department of Medicine, Samsung Medical Center, Sungkyunkwan University School of Medicine, Seoul 06351, Korea; gyeol.seong@gmail.com (G.S.); seungbaek12@gmail.com (S.L.); ys.jang@skku.edu (Y.S.J.); do.chang@samsung.com (D.K.C.); 2 Youdam Co., Ltd. 65, Namsan 2-gil, Jiksan-eup, Seobuk-gu, Cheonan-si 31116, Chungcheongnam-do, Korea; youdam@hanmail.net; 3 Genofocus, Inc., 65, Techno 1-ro, Yuseong-gu, Daejeon 34014, Korea; ejkim@genofocus.com; 4 Laboratory of Pharmacognosy, College of Pharmacy, Dankook University, Cheonan 31116, Korea; soypark23@dankook.ac.kr; 5 College of Biotechnology & Bioengineering, Dankook University, Cheonan 31116, Korea; hichkim@dankook.ac.kr

**Keywords:** irritable bowel syndrome, *Lactobacillus casei*, probiotics, corticosterone, cytokines, tight junction proteins

## Abstract

Non-viable bacteria, referred to as “paraprobiotics,” have attracted attention as potentially safer alternatives to probiotics. The aim of this study was to investigate the efficacy of heat-killed *Lactobacillus casei* DKGF7 on the symptomatic improvement of irritable bowel syndrome (IBS) in a rat disease model and to elucidate the underlying mechanisms that contribute to the beneficial effects of heat-killed probiotics. Seven male Wistar rats were induced with IBS by restraint stress and administered heat-killed *L. casei* DKGF7 for four weeks and then compared with seven rats in the control group. Stool consistency measured four weeks after initial treatment was the primary outcome measure. To investigate the mechanism of action of the heat-killed bacteria on IBS, we measured serum corticosterone levels, inflammatory cytokines in colon tissue, and expression of tight junction proteins (TJPs) in the epithelium. The treatment group showed significantly better stool consistency scores than the control group at week 4, as well as at every measured time point (all *p* values < 0.05). The treatment group showed lower serum corticosterone levels, lower colonic inflammatory cytokine levels, and higher expression of TJPs compared with the control group. Paraprobiotics such as heat-killed *L. casei* DKGF7 can improve stool consistency in a rat IBS model, which may indicate a potential therapeutic strategy for IBS treatment.

## 1. Introduction

A growing body of evidence suggests that gut microbiota play a central role in intestinal diseases and human health [1]. Irritable bowel syndrome (IBS) is a chronic functional bowel disorder with recurrent abdominal pain or discomfort accompanying a change in bowel habits for at least six months [2,3]. The pathophysiology is largely unknown, but recent advances indicate a clear connection between the gut microbiota and intestinal mucosa [4], complex gut–brain disorder [5,6], low-grade mucosal inflammation [7], immune reaction [8], and altered intestinal permeability [9]. This understanding is the basis for treatments, which attempt to maintain a healthy equilibrium between the gut microbiota and host immune response, one of which is the administration of probiotics.

Probiotics are defined by the United Nations Food and Agriculture Organization (FAO) and World Health Organization (WHO) jointly as live microorganisms that benefit the host [10]. By this definition, probiotic bacteria must be alive, and previous studies have suggested that viability and sufficient bacterial load are major requirements for effective probiotic products [11]. However, recent evidence indicates that bacterial viability is not essential for promoting health benefits [12]. Dead bacteria contain cell fragments and retain biological activity that can induce immune responses in the host similar to live bacteria [13,14]. These products have been referred to as “inactivated probiotics” or “ghost probiotics,” but recently the term “paraprobiotics” was coined to describe non-viable or inactivated bacterial cells that, when administered, confer a benefit to the consumer [15].

Paraprobiotics can be obtained through several methods including heating, exposure to ultraviolet rays, and high pressure [16]. *Lactobacillus* are the most widely studied probiotic bacteria [17]; however, little evidence is known about the probiotic effects of heat-killed *Lactobacillus*. Recent studies reported that heat-killed *Lactobacillus* has a protective effect against *Candida albicans* infection in immunodeficient mice [18], as well as immunomodulatory effects such as inducing inflammatory cytokines and expression of tight junction proteins (TJPs) in rats [19,20]. In addition to the beneficial biological activity of heat-killed paraprobiotics, other demonstrated advantages include safety, long shelf life, and convenience for transportation [16].

*Lactobacillus casei* DK207 is one of the strains found to have antioxidant activity isolated from kimchi, a traditional Korean fermented food [21,22]. Recently, this strain was licensed as the code of *Lactobacillus casei* DKGF7 from the Korea health supplement institute (KHSI). In our previous studies, we demonstrated the effect of different species and forms of *Lactobacillus* from kimchi on the improvement of IBS symptoms using a rat IBS model [23,24,25]. Building on that work, this study was designed to evaluate the beneficial effects of heat-killed *Lactobacillus casei* DKGF7 on improving IBS symptoms in an animal disease model and to investigate the mechanisms of action by these paraprobiotics on the disease itself.

## 2. Materials and Methods

### 2.1. Animals and Study Design

**Figure 1:**
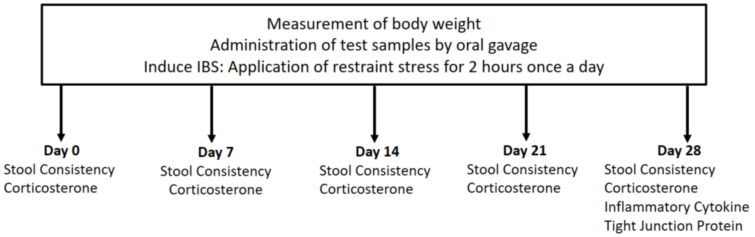
Schematic of this study design.

A restraint model was used to mimic human IBS as described in our previous experiment [25]. Male Wistar rats weighing 304.7 ± 1.4 g and aged eight weeks were purchased from Oriental Bio Co. (Seongnam, Korea) and were raised in a sterile pathogen-free environment. Each animal was housed in a cage maintained at a constant temperature and light-controlled (12 h light and dark cycle), with the same food and water ad libitum. Seven days after acclimatization, the rats were assigned to control and treatment groups (*n* = 7 each) randomly. Both groups were administered oral maltodextrin, and the treatment group was provided with additional heat-killed *L. casei* DKGF7 (1 × 10^11^ CFU) daily for four weeks. All tested rats were immobilized using a close-fitting plastic cage for 2 h daily during the four weeks of the experiment (Figure 1).

Body weight was measured daily for each animal. Stool consistency and serum levels of corticosterone were measured every seventh day over a period of four weeks. The primary outcome for this study was the stool consistency after four weeks. We recorded a three-grade score (0, normal; 2, loose; 4, diarrhea) to evaluate stool consistency. At the end of the experiment, we collected colon tissues to measure expression of inflammatory cytokines and tight junction proteins.

The Institutional Animal Care and Use Committee of the Samsung Biomedical Research Institute (Seoul, Korea) reviewed and approved the study protocol (IRB No. 20200219001). As an accredited facility of the Association for Assessment and Accreditation of Laboratory Animal Care International (AAALAC International), the study abided by the Institute of Laboratory Animal Resources (ILAR) guidelines.

### 2.2. Preparation of the Heat-Killed Bacterial Strain

*Lactobacillus casei* DKGF7 was cultured in 10 mL of MRS broth supplemented with 2% maltose (mMRS) at 30 °C for 24 h. After repeated incubation and cultivation, *L. casei* was centrifuged for 10 min at 5000 rpm. Distilled water was added to achieve a moisture content of 90%, and bacteria were autoclaved at 121 °C for 15 min. After heat-treated preparation, inactivated bacteria was made into powder form and mixed with the maltodextrin. Maltodextrin is an excipient for probiotics sample. Although maltodextrin would have little effect on IBS, we also administered it in control group. Each rat in the treatment group was administered one dose of 500 µL *L. casei* DKGF7 (1 × 10^11^ CFU) daily.

### 2.3. Serum Corticosterone Levels

For measuring serum levels of corticosterone, 0.5 mL of whole blood was obtained weekly from the tail vein of all animals. Serum was separated from whole blood and stored at −80 °C before analysis. Stress hormone concentration was quantified using a corticosterone ELISA kit (Arigo, Hsinchu, Taiwan, China), and the absorbance was measured at 450 nm using an ELISA microplate reader (Thermo Scientific, Waltham, MA, USA). The minimum measurable concentration was 6.1 ng/mL. All experiments were performed in duplicate.

### 2.4. Measurement of Inflammatory Cytokines in Colon Tissues

At the end of the experiment, rats were sacrificed, and the colon tissues were isolated. We measured inflammatory cytokines, including interleukin (IL)-1β, IL-12p70, IL-17A, and tumor necrosis factor (TNF)-α from the colon tissue using the MILLIPLEX MAP Rat Cytokine/Chemokine Magnetic Bead kit (Millipore Sigma, Burlington, MA, USA). The plates were analyzed with a Luminex 100/200 reader using MasterPlex QT2010 software (Luminex, Austin, TX, USA). All samples were tested in triplicate.

### 2.5. Expression of Tight Junction Proteins Using Immunohistochemistry

Tight junction proteins, including claudin-1 and zona occludens-1 (ZO-1), were measured in the colon tissue using immunohistochemistry (IHC) following the protocol of Seong et al. [25]. Briefly, 4-µm paraffin-embedded tissue sections were de-paraffinized with xylene, and endogenous peroxidase activity was blocked with 3% hydrogen peroxide in PBS. The sections were incubated overnight at 4 °C with primary antibodies and then incubated with horseradish peroxidase P-labeled polymer-conjugated antibodies. All IHC slides were scanned using an Aperio ScanScope XT Slide Scanner (Leica, Wetzlar, Germany), and quantitative analysis was performed using ImageJ software.

### 2.6. Statistical Analysis

Comparisons between two groups were conducted using the Mann–Whitney U test and two-way analysis of variance (ANOVA) followed by Bonferroni post-hoc analysis. A *p* value less than 0.05 was considered statistically significant. Data were expressed as mean ± standard error of the mean (SEM) and were visualized using GraphPad Prism 5 software (GraphPad, San Diego, CA, USA).

## 3. Results

### 3.1. Body Weight

**Figure 2:**
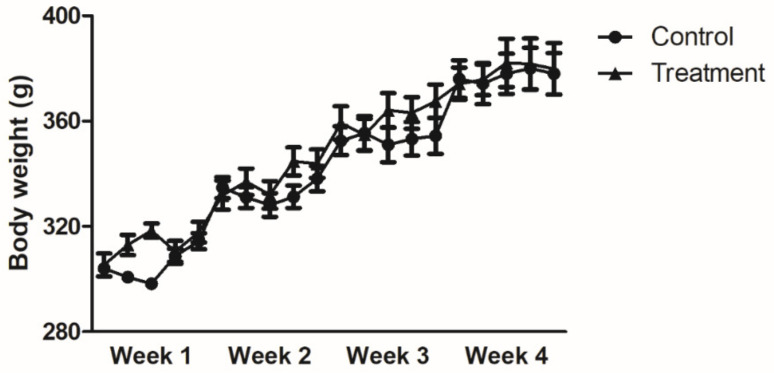
Change in body weight over time. Body weight increased gradually in both treatment and control group. Results are expressed as the mean ± SEM (*n* = 7/group). No significant difference was observed in the body weight between the two groups (two-way ANOVA with post-testing by Bonferonni correction).

Body weight increased gradually during the experimental period in both the control and treatment groups. Four weeks after the start of administration, there was approximately 1.25% weight gain in all subjects (control: 378 ± 7.9 g, treatment group: 380 ± 9.8 g, *p* = NS). However, no significant difference was observed between the two groups (Figure 2).

### 3.2. Stool Consistency

**Figure 3:**
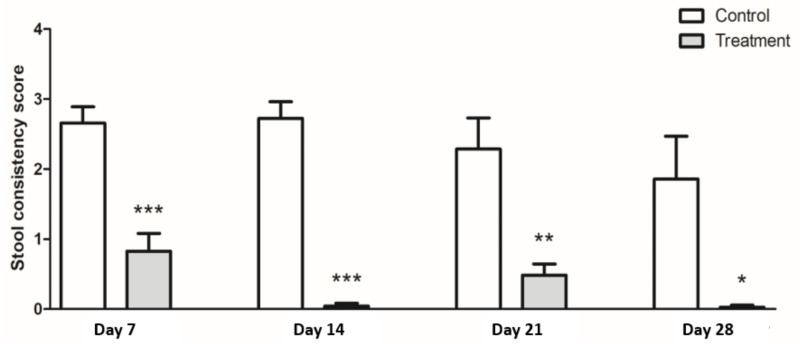
Stool consistency scores. Treatment group (gray bar) showed lower stool consistency score than the control group (white bar). Results are expressed as the mean ± SEM (*n* = 7/group). * Indicates significant p-value differences by an unpaired *t*-test; * *p* < 0.05, ** *p* < 0.01, *** *p* < 0.001.

The treatment group had significantly lower scores for stool consistency than the control group at week 4 (control: 1.9, treatment: 0.4, *p* < 0.05) (Figure 3). This statistically significant difference became apparent by the first week of the study. Although there was fluctuation, all the values of the treatment group were less than 1 for the duration of the study (week 1: 0.83 ± 0.25, week 2: 0.05 ± 0.05, week 3: 0.49 ± 0.6, week 4: 0.03 ± 0.61). Meanwhile, rats in the control group excreted consistently looser stools for the four weeks of the study period and did not recover to normal defecation (week 1: 2.66 ± 0.23, week 2: 2.72 ± 0.24, week 3: 2.29 ± 0.44, week 4: 1.86 ± 0.61).

### 3.3. Serum Level of Corticosterone

**Figure 4:**
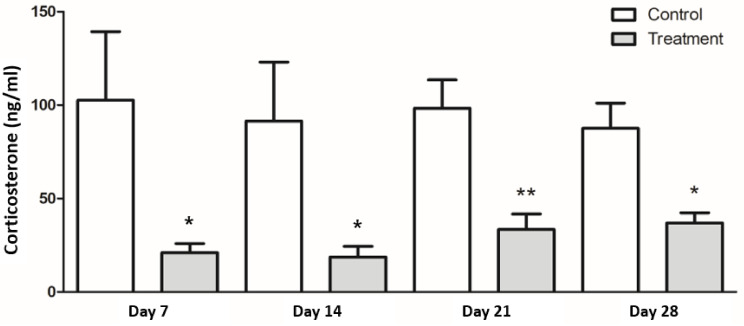
Serum corticosterone levels. Serum corticosterone was measured from blood collected immediately after the restraint period ended at the indicated day. Treatment group (gray bar) showed lower level of serum corticosterone than control group (white bar). Results are expressed as the mean ± SEM (*n* = 7/group). * Indicates significant differences by unpaired *t*-test; * *p* < 0.05, ** *p* < 0.01.

Serum corticosterone levels were significantly lower in the treatment group than in the control group at week 4 (control: 87.6 ± 13.5 ng/mL, treatment: 37.0 ± 5.4 ng/mL, *p* < 0.05), as well as throughout the study period (Figure 4).

### 3.4. Expression of Inflammatory Cytokines in Colon Tissues

**Figure 5:**
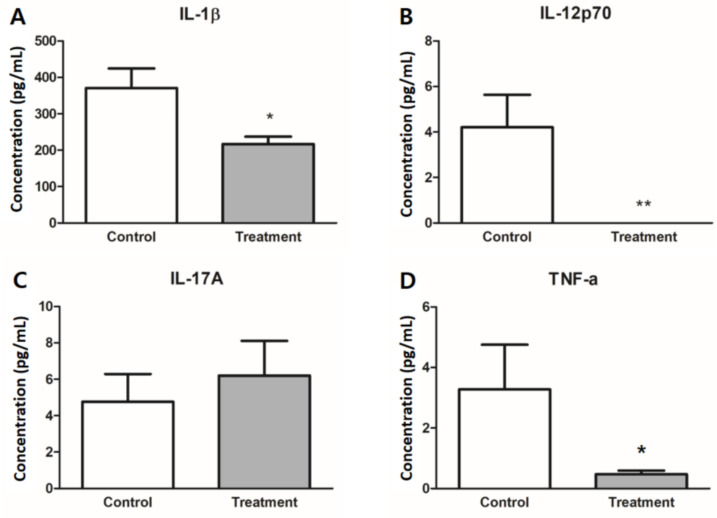
Inflammatory cytokines in colon tissue. Cytokine levels of (**A**) IL-1β, (**B**) IL-12p70, (**C**) IL-17A, and (**D**) TNF-α were measured in colon tissue after the four-week study regimen. IL-1β, IL-12p70, and TNF-α were significantly lower in the treatment group (gray bar) than the control group (white bar). Results are expressed as the mean ± SEM (*n* = 7/group). * Indicates significant differences by unpaired *t*-test; * *p* < 0.05, ** *p* < 0.01.

The treatment group showed lower levels of inflammatory cytokines in colonic tissue, including IL-1β, IL-12p70, and TNF-α (Figure 5). Tissue levels of IL-1β and TNF-α were lower in the treatment group than in the control group (*p* < 0.05). IL-12p70 levels were near the lower detection limit in the treatment group and showed a significant difference from the control group (control: 4.21 ± 1.43 pg/mL, treatment: 0 pg/mL, *p* < 0.01). There was no difference in the IL-17A levels between the two groups.

### 3.5. Expression of Tight Junction Proteins

**Figure 6:**
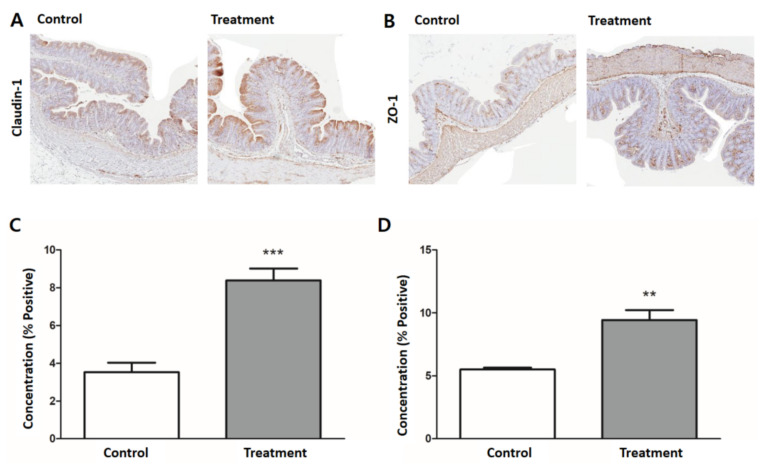
Expression of colonic tight junction proteins. Representative H&E-stained images of colon tissue for (**A**) claudin-1 and (**B**) ZO-1 by immunohistochemistry (200× magnification). Quantification of (**C**) claudin-1 and (**D**) ZO-1 expression were significantly higher in the treatment group (gray bar) than the control group (white bar). Results are expressed as mean ± SEM (*n* = 7/group). ** *p* < 0.01, *** *p* < 0.001.

The treatment group showed significantly higher expression of both claudin-1 and ZO-1. The treatment group showed stronger intensity and wider area of brownish staining in the colonic epithelium. Using ImageJ to scan the slides, the staining intensity of claudin-1, expressed as the ratio of the positively stained area of the entire scanned specimen, was higher in the treatment group than in the control group (control: 3.53% positive, treatment: 8.38% positive, *p* < 0.001) (Figure 6A,C). A similar trend was observed for ZO-1 (control: 5.51% positive, treatment: 9.43% positive, *p* < 0.01) (Figure 6B,D).

## 4. Discussion

In this study, the treatment group administered with heat-killed *L. casei* DKGF7 showed better stool consistency scores than the control group at week 4 and throughout the study period. Additionally, by measuring serum corticosterone, inflammatory cytokines, and tight junction proteins, which represent the stress-induced responses in the intestinal immune system and mucosal permeability, we identified the mechanisms underlying the action of heat-killed *L. casei* DKGF7.

There are several reasons why we focused on non-viable bacteria (“paraprobiotics”) in this study. Concerns have been raised about safety issues with live probiotics such as translocation into systemic circulation, particularly in neonates and immunocompromised patients [26,27,28], persistent colony formation [29], and gene transfer resulting in undesired gain of virulence and resistance to antibiotics [30]. Because probiotic bacteria are meant to be consumed alive, transportation and storage processes are quite strict, and many environmental conditions (e.g., pH, temperature, oxygen, water content) can be an obstacle for bacteria to maintain viability until arrival at the GI tract of the host. Emerging evidence supports the safety of paraprobiotics in vulnerable patients [31]. In terms of pharmaceutical advantages, paraprobiotics have less interaction with food or matrix components, longer shelf life, and greater convenience of administration, thus providing greater favorability than live probiotics [32].

Several investigations have provided explanations as to how dead bacteria can exert biological activity. The main mechanisms underlying the beneficial effects of probiotics are their antimicrobial and immunomodulatory effects [13]. The antimicrobial effect is an interaction between probiotic bacteria and other organisms, including direct inhibition and competition for adhesion sites or nutrients, which is required for viability. The immunomodulatory effect is caused by the interaction between the bacterial cell and the host. In this interaction, the host cell’s ability to recognize bacterial cells plays a more important role than the bacterial viability. Inactivated bacteria are a mixture of dead cells, cell wall structures, cell-free supernatants, and metabolites, which can be immunologically effective in live bacteria [15]. Heat-killed *Lactobacillus rhamnosus* GG was able to downregulate TNF-α-induced IL-8 production through activation of the nuclear factor κB (NFκB) pathway [33]. In addition, while live microorganisms in the mucosal layer are rarely able to directly attach to the epithelium and immune cells because of the mucosal barriers, microbial metabolites can pass through the gut mucosal barrier more easily and directly stimulate the host’s epithelial cells and macrophages [34,35].

In addition to being an immunomodulatory effector, heat-killed bacteria can serve as an enhancer of mucosal barrier function. Heat-killed bacteria and their cell components have protective effects against pathogenic bacteria. Exopolysaccharides on the bacterial cell surface can prevent adhesion and biofilm formation of pathogenic bacteria [36], and components of xyloglucan or gelatin tannate fortify mucosal barrier function by interfering with pathogenic bacteria [37]. Another study reported that a heat-killed paraprobiotic mixture could increase intestinal mucosal barrier integrity by regulating the expression of the tight junction protein ZO-1 [38].

The pathophysiology of IBS is associated with altered gut–brain interaction, activation of the immune system, and gut barrier dysfunction, leading to increased intestinal permeability [5]. Corticosterone is a major hormone that plays an important role in the hypothalamic–pituitary–adrenal (HPA) axis as a link between the brain and the intestinal immune system. The HPA axis is overactivated in IBS patients [39], and corticosterone mediates stress-induced increased mucosal permeability [40]. In response to psychological stressors such as anxiety and depression, pro-inflammatory cytokines increase in the colonic mucosa and are released into the serum [41]. High levels of inflammatory cytokines such as IL-1β and TNF-α [8,42] and low expression of TJPs such as claudin-1 and ZO-1 have been observed in IBS patients with diarrheal symptoms [43].

There is a considerable amount of data demonstrating the beneficial effects of probiotics on improvement in IBS [25,44] and the ability of non-viable bacteria as an immunomodulator in the intestinal environment of hosts [15]. However, there is still a lack of evidence supporting the clinical use of heat-killed bacteria as a treatment for IBS. In this study, the treatment with heat-killed *L. casei* DKGF7 showed lower levels of serum corticosterone, lower inflammatory cytokines in colonic tissue, and higher expression of TJPs in intestinal mucosa, compared with the control group. It could be inferred that heat-killed *L. casei* DKGF7 may have immunomodulatory effects, thus improving mucosal integrity, leading to improvement of IBS symptoms.

There are several considerations for this study. Heat-killed treatment is the most widely used method for inactivating probiotic bacteria. It has been shown to cause less damage to the cell surface than the high-pressure method [45], and is associated with optimal pharmaceutical advantages including long shelf lives [32]. Nevertheless, the immunogenic properties influenced by inactivation methods and bacterial strains used requires careful consideration [45,46]. Paraprobiotics can be obtained through various methods such as heating, administration of high pressure, exposure to ultraviolet radiation, and sonication [16], and immunomodulatory properties are affected by the mode of inactivation [47]. Even in thermal treatment, the beneficial effects depend on the activation conditions, such as temperature and time. High temperatures are associated with a greater degree of coarseness of the cell surface and decreased adhesion capacity [48]. Therefore, more detailed evidence about how different types of inactivation influence the bacterial structure and its beneficial effects are needed. Additionally, further studies are required to compare different strains, the same strains of live versus dead bacteria, and exact immunologic targets.

Another limitation of this experiment is the use of the restraint stress model in rats. Restraint stress can cause visceral hypersensitivity and colonic hypermotility, leading to aggravation of stool consistency in rats [49]. Rats exposed to restraint stress showed a lower threshold of corticotropin releasing factor (CRF), hyper-responsiveness to CRF [50], and increased intestinal permeability [51]. Although the IBS rat model cannot completely reflect human IBS, there are similarities between the intestinal response of the wrap-restraint model and stress-related IBS symptoms in humans. Therefore, the restraint stress rat model is considered to be appropriate for evaluating the effect of heat-killed bacteria in IBS [52]. However, we could not assess important characteristics of IBS including abdominal pain and pain relief after defecation, due to the inherent limitations of animal models. Further investigations are warranted to evaluate the various IBS symptoms in patients with IBS. As the aspect of experimental design, since non-IBS control was not set up in this study, we could not see whether the treatment group improved toward non-IBS or pre-induction mice. In addition, we were able to observe that the treatment group with heat-killed L.casei had less induced IBS symptoms than the untreated group; however, we could not reveal whether heat-killed L.casei could treat the mice with pre-existing IBS into healthy status.

In conclusion, we observed that heat-killed *L. casei* DKGF7 has beneficial effects on symptom improvement in an animal IBS model and the underlying mechanisms of disease. Paraprobiotics derived from heat-killed bacteria may have potential as an alternative therapeutic strategy in IBS.
